# Study on Balance and Postural Control According to the Stabilometry in Indoor Skydivers: A Cross-Sectional Study

**DOI:** 10.3390/ijerph20010635

**Published:** 2022-12-30

**Authors:** Carlos López-de-Celis, Daniel Zegarra-Chávez, Aïda Cadellans-Arróniz, Andoni Carrasco-Uribarren, Pedro Izquierdo-Nebreda, Max Canet-Vintró, Jacobo Rodríguez-Sanz, Albert Pérez-Bellmunt

**Affiliations:** 1 Faculty of Medicine and Health Sciences, Universitat International de Catalunya, 08028 Barcelona, Spain; 2 ACTIUM Functional Anatomy Group, 08028 Barcelona, Spain; 3 Fundació Institut, Universitari per a La Recerca a l’Atenció, Primària de Salut Jordi Gol i Gurina (IDIAPJGol), 08028 Barcelona, Spain

**Keywords:** indoor skydivers, postural balance, stabilometry, posturographic, posture

## Abstract

Background: The wind tunnel is a compression cabin through which a stream of air rises homogeneously, generated by fans. To perform different acrobatics, indoor skydivers have to change their body position by turning their body and orientation in reference to the space. Thus, the vestibular, visual and somatosensory systems are subjected to multiple disturbances. Postural control could be affected by altering the visual, vestibular and somatosensory systems during indoor skydiving in the wind tunnel. The aim of this study is to describe the influence of a standard wind tunnel training session on postural control in a normal gravitational situation in indoor skydiving. Methods: Ten indoor skydivers registered with the Royal Spanish Aeronautical Federation, who had participated in national or international competitions one year ago, were recruited. A single 30 min training session was performed. Postural control was assessed through posturographic analysis using a stabilometric platform immediately before and after the training session. The variables studied were related to the relative position and length of the centre of pressure. Results: No statistically significant changes were found between the initial and final assessment for the posturographic variables studied. Conclusions: No differences in postural control were found after a standard wind tunnel training session in indoor competition skydivers.

## 1. Introduction

The first parachute was created by Leonardo da Vinci in the 15th century. For a long time, the practice of skydiving was only carried out by skilled military personnel. However, today civilians can practice the sport regularly. Several simulators have been designed to learn and improve freefall-related skills, including virtual skydiving simulators and wind tunnels [1]. The wind tunnel is a vertical compression cabin within a cylindrical space, with a conical upper part through which a stream of air generated by fans rises homogeneously [2]. These powerful fans allow people to free fall in different flying positions. The indoor skydiver can exceed speeds of 300 km/h in freefall and reach three meters in height during a normal training session, although depending on the flying chamber, a greater height can be achieved by the flyers [3]. The first recreational wind tunnel was built in 1979 in Las Vegas (USA) to develop individual and group training drills, which could minimise risk and costs. Petruck VB [1] notes that using the wind tunnel could optimise skydiving training for the military and civilians.

To our knowledge, there is a lack of studies focusing on wind tunnel injuries. The literature reviewed shows a single case of acute injury to the musculocutaneous nerve due to the maintained position of abduction, extension and external rotation of the upper limb [4]. In addition, an acute vertebral pain trend related to posture has been described [5]. In that sense, forced hyperextension posture supported by skydivers can cause discomfort and alterations in the spine, as previously reported.

In the wind tunnel, skydivers focus on two positions: head up and down. They must work alongside the wind to perform body orientation changes, turns and stunts. During this sport, the vestibular, visual and somatosensory systems are subjected to multiple disturbances. Therefore, it is necessary to have optimal postural control to adapt to each situation and to be able to perform these acrobatics correctly. Postural control is based on the information captured by the visual, vestibular and somatosensory systems that configure the strategies to maintain balance in the multiple actions of daily life through brain organisation and muscular synergies [6]. Therefore, postural control could be affected by disrupting the visual, vestibular and somatosensory systems during indoor skydiving in the wind tunnel. Constant orientation changes could alter the vestibular and visual systems. The information received by the somatosensory system could be altered by spinal irritation, as has been documented in the literature [5,7]. In addition, the subject does not have a stable support base to lean on (the balance point is the wind, whose speed reaches 180–300 km/h). All of this could alter the athlete’s postural control and balance.

One of the tools used to assess postural control and balance is the stabilometric platform, which performs a stabilometric analysis of the centre of pressure using a force platform [8]. The parameters provided by these devices that characterise postural control are the trajectory of the centre of pressure, the surface area in cm^2^ projected by the centre of pressure on the force platform and the coordinates on the X and Y axes. The parameters above estimate the contribution of the visual system in postural control [9].

The literature has described stabilometric characteristics in different sporting disciplines that can alter postural control, such as taekwondo, tennis [10], gymnastics [11] and ballet [12] or in subjects’ vestibular, visual or somesthetic system alterations [13,14]. Astronauts exposed to microgravity have also shown alterations in balance and upright posture [15]. However, whether balance and postural control changes occur in indoor skydivers during their sport has yet to be discovered. Knowing these parameters will allow us to determine if there are any benefits to balance and postural control within this sport. This study aimed to describe the influence of a standard training session on postural control in the wind tunnel in indoor skydivers.

## 2. Materials and Methods

### 2.1. Study Design

A descriptive cross-sectional study was conducted. The local ethics committee approved the study protocol (CBAS-2019-15). The procedures followed the Declaration of Helsinki 1975, Fortaleza 2013. The study was conducted at the Windoor events facilities (Windoor, Empuriabrava and Barcelona). All research was reported in accordance with STROBE guidelines/regulations. Informed consent was obtained from all participants.

### 2.2. Subjects

We recruited ten dynamic indoor skydivers federated in the Royal Spanish Aeronautical Federation, taking advantage of pre-competition training days between January and February 2021. The recruited sample represents 91% of the total number of registered indoor skydivers in Spain.

The inclusion criteria were: (1) Being an indoor skydiver registered with the Royal Spanish Aeronautical Federation. (2) Participated in national or international competitions in the last year. (3) Signed the informed consent for participation in the study. The exclusion criteria were: (1) Presenting a history of orthopaedic injuries in the last six months that could influence their postural control or balance capacity (ankle sprain, lower limb fracture). (2) Presenting a medical diagnosis of vestibular disorders at the time of the study.

### 2.3. Measurements

The descriptive and anthropometric variables age, sex, hours of sports practice, height, weight, body mass index, foot length (distance between the heel and the tip of the big toe or the tip of the second toe in cases where the latter is longer) and measurement on the strength platform were recorded.

The posturographic variables studied were surface area (mm^2^), which comprises 95% of all measured points of the centre of pressure and gives information on stability. The variables that show the positional point in the ellipse in the X and Y coordinate axes and that evaluate the symmetry of the postural position were the Mean_X value (mm) and Mean_Y value (mm). Furthermore, the total length (mm) represents the precision of the postural system in maintaining balance; finally, the variables length_X (mm) and length_Y (mm) show the distance covered by the centre of pressure on the abscissa and ordinate axis.

The results were measured at baseline and immediately after a standard training test (post-jump indoor). The stabilometric platform Satel 40/16 was used for this purpose, together with the Satel v 33.5 8C (r = 0.9–1.0) [8] posture-kinetic activity evaluation programme.

Data collection was carried out at the Windoor events facilities (Empuriabrava—Girona and Cornella de Llobregat—Barcelona). Once the selection criteria had been checked and the informed consent form had been signed the descriptive and anthropometric variables were recorded. Before the training session, a posturographic assessment was performed. The assessment room was located as close as possible to the tunnel’s exit to avoid displacement or other confounding factors. All participants wore noise protection.

**Figure 1:**
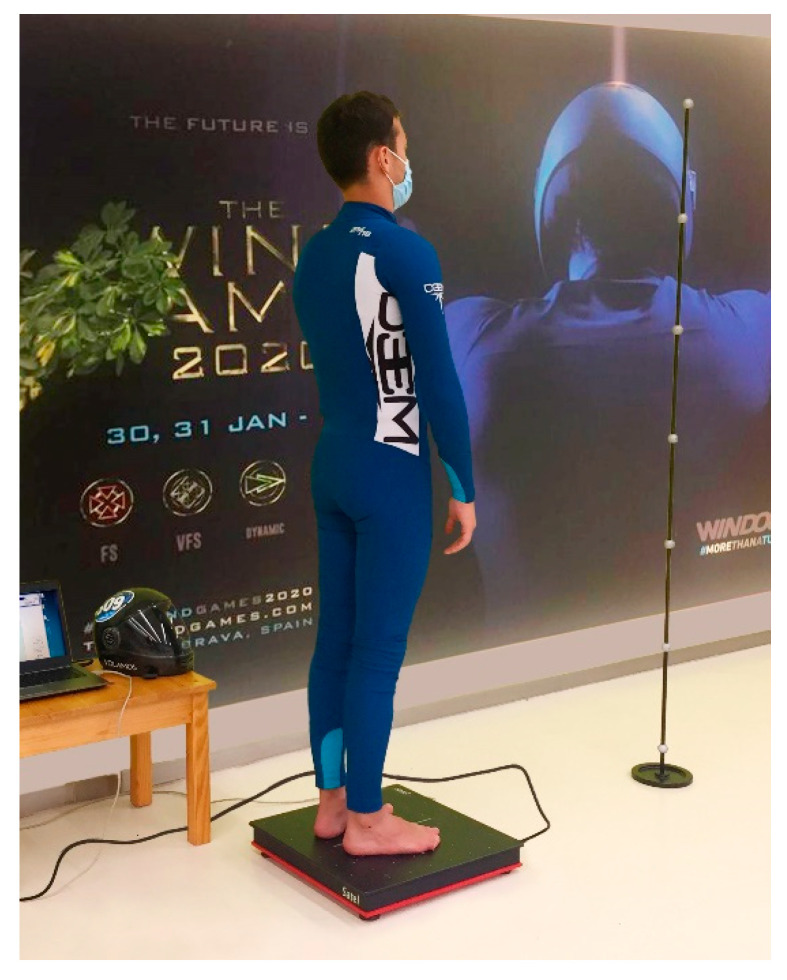
Indoor Skydiver placed in a bipedal position on the platform.

The protocol for the stabilometric measurement followed in the study was according to “Norms 85”, established by the French Posturology Association [16]. In all measurements, the subject remained standing on the platform without shoes or socks. The subject was placed in a bipedal position on the platform with the heels 2 cm apart, using a foot positioning template. The insole allows correct positioning of the feet at an angle of 30 degrees and maintains the bisector with the sagittal axis of the platform. The platform was placed 90 cm from the wall, where a vertical reference was placed as a visual reference for measurements taken with the eyes open [8]. When we had a correct position of the subject, a verbal command was given to keep the arms parallel to the body and to maintain the position, avoiding any movement [8] (Figure 1). The recording was performed in a bipedal posture with eyes open (OA) (52 s), followed by eyes closed (EC) (52 s). The exact sequence was repeated twice [12,17].

### 2.4. Standar Trainng Session

A standard session consisted of 30 min of training time, with periods of 2.30 min of flying and 2.30 min of rest. During the rest periods, the participants swapped with other participants, remaining in a standing position without performing any other activity. In total, 15 flights were made.

**Figure 2:**
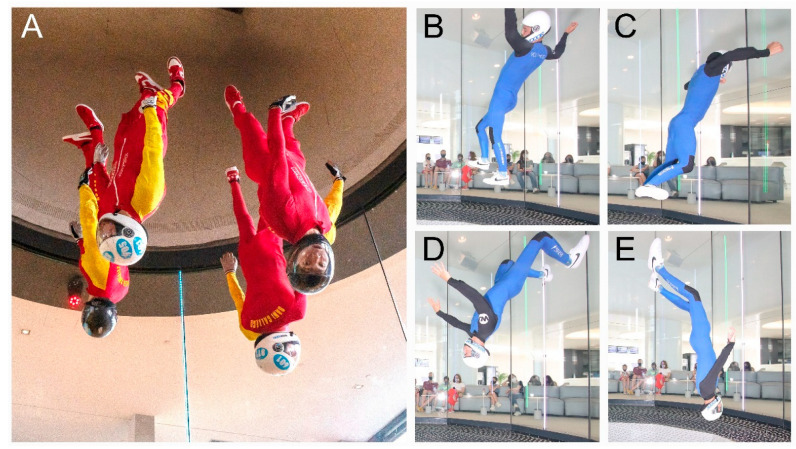
(**A**) Indoor Skydivers from Royal Spanish Aeronautical Federation. (**B**) Head-up in-face position. (**C**) Head-up out-face position. (**D**) Head-down in-face position. (**E**) Head-down out-face position.

Different figures and synchronized crossings were practiced during the training, which we could mainly differentiate into four positions. Head-up: the athlete performs the acrobatics in an upward-facing position; head-down: the athlete performs the acrobatics facing downwards; in-face: the athlete performs the manoeuvres facing the inside of the wind tunnel; out-face: the athlete stunts are performed facing the outside of the wind tunnel (Figure 2).

### 2.5. Statistical Analysis

For statistical analysis, IBM SPSS Statistic 26.0 software was used. Descriptive analysis was carried out. For quantitative variables, mean and standard deviation were calculated. Frequencies were calculated for demographic and anthropologic qualitative variables. Normality distribution was assessed using the Shapiro–Wilk test. The Wilcoxon test was used to analyse differences between baseline and post-flight. Effect sizes were calculated using Cohen’s d coefficient. An effect size > 0.8 was considered large; around 0.5, intermediate; and <0.2, small [18]. The significance level was *p* < 0.05.

## 3. Results

Between January and February 2021, 10 volunteers were recruited (9 males, 1 female), with a mean age of 27.2 years (SD 2.3). The demographic characteristics of the sample are summarized in Table 1.

We analysed whether there was a difference between the sessions, finding that the length and surface values decreased with eyes open while they increased with eyes closed. In none of the variables analysed a statistically significant difference was found. Furthermore, the effect size with Cohen’s d was between small and intermediate for all variables (Cohen’s d between: 0.03–0.41), with all variables being lower in the eyes closed assessment, except for the surface variable, which was higher (Table 2).

## 4. Discussion

This study aimed to describe the influence of a standard training jump in postural control inside the wind tunnel in indoor skydivers. The analysed subjects performed a balance test on a stabilometric platform before and after the standard training session. The results indicated no statistically significant differences between postural control ability before and after training in a population of trained indoor skydivers. Previous studies have analysed the stabilometric characteristics in sports where optimal control is fundamental for their discipline [10,11,12]. Ozdemir et al. showed altered balance and upright position after gravity alterations [15]. However, to our knowledge, this is the first research to assess the effect of indoor flying on postural control.

The tool used for the analysis was the stabilometric platform, whose reliability was validated and analysed by Rodríguez-Rubio et al. in a sample of healthy university subjects [8]. The subjects they analysed were healthy and younger (20.98 ± 1.83) than our group of indoor skydivers (27.20 ± 4.76). However, the parameters recorded show a worse initial balance than our sample. The sample of the study by Rodríguez-Rubio et al. does not detail whether the subjects included practiced any sport discipline, which may justify this difference in postural control ability [8]. It is hypothesised that physically active subjects have better balance, which is why balance training is recommended in some cases to improve athletes’ skills [19]. However, some studies, such as that of Janura et al., concluded that professional dancers did not show better balance than non-professional dancers [12].

It is observed that after standard training in the wind tunnel, both with eyes open and eyes closed, the subjects showed no difficulties performing the balance test on the stabilometric platform. Although some parameters were somewhat worse after training during the standard session, mainly the eyes-closed variables, the parameters remained practically stable. The minor changes in postural control suggest that athletes trained in this discipline prioritise information from one or other systems depending on the situation in which they find themselves. This type of neuronal plasticity has already been described in other populations, for example, in astronauts who, after a period in microgravity, prioritise information from the visual and somesthetic systems, reducing the importance of the vestibular system. This neural plasticity has also been described in people who work standing up on a train. These people endure constant vibration that could alter the somesthetic system, with continuous acceleration and deceleration that could alter the information of the otic vestibular system, added to the continuous changes supported by the visual system (changes of scenery, changes of light due to tunnels, etc.). This population shows optimal balancing capacity, probably due to the ability to collect information from each system more optimally, prioritising each system’s information according to the situation [20,21,22]. As discussed above, balance and postural control depend on the correct integration of information provided by the vestibular, ocular and somesthetic systems [6,23]. We assume that balance systems are stressed during a standard training session in the wind tunnel, leading us to hypothesise that postural control disturbances may occur after the flight. Surprisingly, our results would suggest the opposite. This study shows no effect on balance and postural control in trained indoor skydivers in the stabilometric assessment. However, comparing the results with untrained subjects with similar characteristics to those of this sample is necessary. We can then clarify whether the lack of change is due to the benefit of training or whether there is no effect on balance in this type of sport.

This study has some limitations. The main one is that as it is a minority sport and in trained athletes, our sample size was small, so we cannot ensure that the results are the same in an amateur population. Another limitation is focused on reproducing the training protocol, which is why we have named the central figures that the subjects practised. On the other hand, it was not feasible to carry out the study in a room with a low or no noise level, so we made sure that there were no noise distractions and that it was stable during the measurements.

## 5. Conclusions

No relevant differences in postural control were found before and after a standard wind tunnel training jump in indoor competition skydivers.

## Figures and Tables

**Table 1:** Descriptive values of the sample.

	n (%) or Mean ± SD
Gender	
Male	9 (90%)
Female	1 (10%)
Age (years)	27.20 ± 4.76
Foot length (cm)	44 ± 2.17
Height (cm)	178 ± 9
Weight (Kg)	73.10 ± 11.27
BMI	23.23 ± 3.22

Abbreviations, n: Number, SD: standard deviation, cm: centimeter, Kg: Kilograms.

**Table 2:** Pre- and post-jump indoor descriptive values and the difference.

	Baseline	Post-Jump Indoor	Difference between Baseline and Post-Jump Indoor (95% CI)	*p*	ES
EO Length (mm)	476.24 ± 102.50	441.61 ± 109.53	34.63 [−59.8; 129.0]	0.241	0.33
EO Length_X (mm)	284.73 ± 65.29	259.29 ± 57.77	25.44 [−22.7; 73.6]	0.241	0.41
EO Length_Y (mm)	322.12 ± 73.69	301.44 ± 95.73	20.68 [−58.7; 100.0]	0.241	0.24
EO Surface (mm^2^)	247.14 ± 121.70	217.63 ± 81.73	29.51 [−40.1; 99.1]	0.799	0.29
EO Mean_X (mm)	1.98 ± 6.82	3.03 ± 6.79	−1.05 [−4.5; 2.4]	0.575	0.15
EO Mean_Y (mm)	−41.13 ± 14.71	−38.61 ± 14.20	−2.52 [−11.3; 6.2]	0.445	0.17
EC Length (mm)	601.86 ± 179.39	640.00 ± 210.77	−38.14 [−171.4; 95.1]	0.959	0.20
EC Length_X (mm)	346.76 ± 108 48	367.12 ± 119.74	−20.36 [−75.8; 35.0]	0.508	0.18
EC Length_Y (mm)	418.14 ± 129.33	444.99 ± 168.20	−26.84 [−147.4; 93.7]	0.799	0.18
EC Surface (mm^2^)	281.02 ± 216.22	288.68 ± 143.26	−7.67 [−121.6; 106.3]	0.203	0.39
EC Mean_X (mm)	2.59 ± 5.59	3.25 ± 7.76	−0.66 [−5.5; 4.2]	0.646	0.10
EC Mean_Y (mm)	−41.07 ± 15.25	−40.58 ± 13.44	−0.49 [−6.7; 5.7]	0.799	0.03

Abbreviations, EO: eyes open, EC: eyes closed, mm: millimetres, mm^2^: millimetres squared, ES: Effect size.

## Data Availability

The data presented in this study are available on request from the corresponding author.
